# How mindfulness influences restrictive eating through the mediation of body image: a diary report study

**DOI:** 10.3389/fpsyg.2025.1547354

**Published:** 2025-05-30

**Authors:** Kequn Chu, Jing Ge, Huan Fan, Qinyi Wu

**Affiliations:** ^1^College of Educational, Guangxi Science & Technology Normal University, Laibin, China; ^2^Faculty of Public Health, Guilin Medical University, Guilin City, Guangxi Province, China; ^3^Department of Basic Science, Faculty of Health Science, University of Technology MARA, Selangor, Malaysia

**Keywords:** state mindfulness, trait mindfulness, restrictive eating, body image, mediation effect

## Abstract

**Objective:**

This study investigated whether body image mediates the link between mindfulness (state/trait) and restrictive eating behaviors.

**Methods:**

A 6-day daily survey of 65 females with restrictive eating patterns incorporated correlation, regression, and multilevel mediation analyses.

**Results:**

Trait mindfulness, state mindfulness, and body image were positively interrelated, while all three demonstrated inverse relationships with restrictive eating. Both state and trait mindfulness predicted reduced restrictive eating behaviors. Critically, body image fully mediated the effect of state mindfulness on restrictive eating at the intra-individual level, suggesting that daily improvements in mindfulness enhance body image, which subsequently reduces maladaptive eating.

**Conclusion:**

State mindfulness mitigates restrictive eating by fostering positive body image, highlighting body image's role in mindfulness-based interventions. Further research should validate this pathway across diverse populations.

## Introduction

Restrictive dieting refers to the tendency to control food intake by resisting internal cues (such as hunger or satiety) and external cues (such as caloric value and palatability) as a means to manage body weight (Martins et al., 2009; Dohle and Hartmann, 2023). It is identified as a critical maladaptive behavior in the progression from excessive concern over body weight and shape to the development of eating disorders (Kong et al., 2011). Compared to non-restrictive eaters, individuals who engage in restrictive dieting are paradoxically more prone to overeating (Houben et al., 2010). This cycle of repeated dieting and overeating not only fails to achieve the desired effects but may also provoke negative emotions such as inferiority and anxiety (Hartley et al., 2018), leading to adverse behaviors and even suicidal tendencies (McGrath, 2012). Experimental evidence indicates that restrictive eating behaviors can predict the severity of eating disorders (Johnson and Wardle, 2005; Lloyd et al., 2020) and constitute a risk factor for the development of bulimia nervosa (Kelly et al., 2011). Furthermore, researchers have found that individuals who engage in restrictive dieting exhibit dulled interoceptive awareness (Shen, 2016) and demonstrate attentional biases when processing body weight, body shape, and food-related information (Kong, 2012; Hollitt et al., 2010).

Synthesizing previous research, the factors influencing the eating behaviors of restrictive dieters can be categorized into three main types: (1) psychological factors related to the restrictive dieters themselves, such as attitudes toward body shape, cognitive biases toward food, emotions, motivations, attention, goals of restrictive dieting, and self-regulatory capacity; (2) factors related to food, such as caloric content and palatability; (3) factors related to the eating environment, such as the location of eating, the presence of others during meals, and media representations of food (Contento et al., 2005). Among these, the most fundamental factors influencing restrictive eating behavior are the psychological aspects of the restrictive dieters themselves (Kong et al., 2011; Wang and Chen, 2019; Zhou et al., 2012). The purpose of this study is to investigate how mindfulness (both state and trait) influences restrictive eating behaviors through the mediating role of body image, utilizing a diary design to capture dynamic within-person processes. By clarifying this mechanism, we aim to inform targeted mindfulness-based interventions (MBT) for reducing maladaptive eating patterns among female college students.

Mindfulness-Based Interventions (MBT) have led to improvements in patients' self-acceptance and self-compassion, thereby enhancing treatment outcomes (Jordan et al., 2014; Rodríguez et al., 2014; Tran et al., 2020). Since restrictive eating is a pivotal stage in the development of eating disorders, mindfulness-based interventions are likely to have a positive effect (Kao et al., 2025).

Mindfulness can be viewed from two perspectives: state mindfulness and trait mindfulness (Duan, 2014). State mindfulness is an individual's ability to focus attention entirely on the present moment, with variability among individuals. This state can be cultivated or altered through meditation practice (Ruimi et al., 2022). Trait mindfulness, on the other hand, refers to an individual's enduring tendency to approach present experiences and events with an accepting and clear-headed attitude (Mercogliano, 2024). It is considered character strength inherent in the individual. Research indicates that individuals with high levels of mindfulness evaluate stress more positively, employ more effective stress-coping strategies, experience lower levels of anxiety and neuroticism, are more optimistic, and report greater wellbeing (Weinstein et al., 2009). Examining both state and trait mindfulness allows us to capture dynamic fluctuations and stable tendencies in mindful awareness.

Meta-analytic results suggest that mindfulness has extensive benefits on individual health (Querstret et al., 2020; Grossman et al., 2004). According to the Theory of Embodiment (Levy, 2022), bodily sensations significantly affect individual behaviors and psychology. Individuals with eating disorders tend to neglect internal physiological signals and have weak interoceptive abilities, making them less sensitive to these signals and gradually forming unhealthy eating behavior tendencies. State mindfulness helps individuals become more aware of and accept their current bodily sensations, respond naturally to physiological signals, and thereby develop positive bodily experiences, alleviating symptoms of eating disorders such as anorexia nervosa (Rodríguez et al., 2014). Additionally, individuals with high levels of trait mindfulness exhibit less impulsive eating and are more inclined to choose healthy snacks (Jordan et al., 2014), with fewer restrictive eating behaviors (Zhao and Liu, 2017). Thus, it is inferred that individuals with high trait mindfulness have better eating habits.

Body image refers to an individual's evaluation and emotional experience of their own body, which can change over time and across situations (Cash et al., 2002). It is a component of self-concept. Previous research has found that trait mindfulness can predict positive self-concepts, as it is closely related to self-esteem, self-efficacy, self-control, and core self-evaluation (Greason and Cashwell, 2009; Kong et al., 2014). Individuals lacking state mindfulness tend to engage in habitual negative self-evaluation and often struggle to accept themselves, which prevents them from focusing on the tasks at hand (Verplanken et al., 2007). Previous research has shown a significant positive correlation between mindfulness and positive body image (Al-Ghabeesh and Mahmoud, 2022; Lavender et al., 2012).

Negative body image is a key factor contributing to restrictive eating behaviors (Gerner and Wilson, 2005). According to the Theory of Planned Behavior, an individual's attitude toward a specific behavior, subjective norms, and perceived behavioral control all determine their intention to act (Ajzen, 1991). From the perspective of attitudes toward restrictive eating behaviors, individuals dissatisfied with their body shape place greater importance on physical appearance and the value of maintaining weight, thus adopting a positive attitude toward restrictive dieting (Cash et al., 2002). From the angle of subjective norms, factors such as teasing by family members about appearance, maternal dieting behaviors, and media portrayals can lead individuals to develop body image distress (Liang et al., 2017). Consequently, individuals with a negative body image are more susceptible to perceiving dietary control as a subjective norm influenced by social and environmental factors. Regarding perceived behavioral control, restricting one's diet is a relatively low-difficulty task that does not require cooperation from others, thus fostering a strong sense of control. Moore's study confirmed that individuals dissatisfied with their body shape are more inclined to attempt body weight control through restrictive eating, self-induced vomiting, and the use of laxatives (Moore, 1988). Therefore, this study posits that a negative body image is more likely to result in restrictive eating behaviors due to the influence of these three aspects. Additionally, Kelly found that a positive body image helps reduce eating disorders (Kelly and Stephen, 2016); suggesting that a positive body image may also decrease restrictive eating behaviors. Female college students were selected for this study because they are more susceptible to body image problems and restrictive eating behaviors in a socio-cultural context that emphasizes thinness (Hamid et al., 2023). This study aims to explore the relationships among mindfulness, body image, and restrictive eating behaviors in female college students.

Given the significant differences in definitions within the two perspectives of mindfulness, and the fact that most current research focuses on trait mindfulness with limited studies examining state mindfulness, it is important to note that state mindfulness can reveal fluctuations in consciousness and their impact on behavior. Therefore, to comprehensively analyze the effect of mindfulness on eating behaviors, this study considers both levels of mindfulness. It explores the impact of state mindfulness at the intra- and inter-individual levels on body image and restrictive eating behaviors, and the effect of trait mindfulness at the inter-individual level on body image and restrictive eating behaviors. In summary, the objectives of this study are twofold: first, to examine the influence of trait and state mindfulness on restrictive eating behaviors, and second, to determine whether body image mediates the effects of different levels of mindfulness on restrictive eating behaviors. Based on these objectives and the theoretical framework outlined above, we propose the following hypotheses:

H1a: State mindfulness negatively predicts restrictive eating.

H1b: Trait mindfulness negatively predicts restrictive eating.

H2a: Trait mindfulness positively predicts body image.

H2b: State mindfulness positively predicts body image.

H3: Body image negatively predicts restrictive eating.

H4: Body image mediates the relationship between mindfulness (state/trait) and restrictive eating.

## Methods

### Participants

Participants were recruited from two public universities in Guangxi, China, selected based on their accessibility and representative student demographics in southern China. Recruitment was conducted through online announcements (university portals, WeChat groups) and in-person flyers distributed across campus public areas (e.g., libraries, cafeterias) to minimize selection bias.

Initially, 415 female students completed the Dutch Eating Behavior Questionnaire (DEBQ) screening. Restrictive eaters were defined as those scoring ≥3 on the DEBQ Restrained Eating subscale (Van Strien et al., 1986; Wang, 2012). Of the 114 identified restrictive eaters, 2 participants were excluded due to BMI >23.00 (classified as overweight according to Chinese standards; Chen et al., 2004), and 47 were excluded for incomplete daily reports (<50% completion rate). The final sample comprised 65 female students (age range: 17–24 years, *M* = 19.32, *SD* = 1.93; BMI range: 18.50–22.90, *M* = 20.34, *SD* = 3.05). Eighty five percentage reported middle-class family income (monthly household income: ¥5,000–20,000).

The sample size was determined based on simulations for multilevel mediation models (Zhang et al., 2016), which recommend ≥50 participants with ≥3 repeated measures to detect medium-sized effects (80% power). *Post-hoc* power analysis via GPower 3.1 (Faul et al., 2007) confirmed 85% power for medium effects with a medium effect size (*f*^2^ = 0.15). Our design (*N* = 65, 5.22 daily entries per person) meets these thresholds.

### Instruments

#### Dutch Eating Behavior Questionnaire

The Dutch Eating Behavior Questionnaire (DEBQ) was originally developed by Van Strien et al. (1986). We used the Chinese version revised by Wang (2012). Specifically, the study focused on the Restrained Eating subscale to identify participants who practice restrictive eating. This subscale includes 10 items, such as “How often do you try not to eat at night because you are dieting?” and utilizes a 5-point Likert scale, ranging from 1 (“never”) to 5 (“always”). Scores are calculated by averaging the responses, with higher scores indicating a stronger propensity for restrictive eating behaviors. In this study, the internal consistency coefficient of the restricted eating portion scale was 0.94.

#### Mindful Attention Awareness Scale

The Mindful Attention Awareness Scale (MAAS) was developed by Brown and Ryan (2003). The Chinese adaptation was validated by Chen et al. (2012). The MAAS features a unidimensional structure with 15 items, such as “I could be experiencing some emotion and not be conscious of it until some time later.” It employs a 6-point Likert scale, where 1 signifies “always” and 6 signifies “never,” with higher scores reflecting greater levels of trait mindfulness. In this study, the MAAS demonstrated an internal consistency coefficient of 0.86.

Additionally, The State Mindful Attention Awareness Scale (MAAS-S) was adapted from the MAAS (Brown and Ryan, 2003). The Chinese version was modified by Chen et al. (2012). The State Mindful Attention Awareness Scale (MAAS-S) was used to measure state mindfulness. This scale is also unidimensional and comprises five items, including “I found it difficult to stay focused on what was happening at the present moment today.” It uses a 6-point Likert scale, similar to the MAAS, where a score of 1 means “always” and a score of 6 means “never.” Higher scores indicate greater state mindfulness. In this research, the MAAS-S showed an internal consistency coefficient of 0.90.

#### Body Image States Scale

The Body Image States Scale (BISS) was developed by Cash et al. (2002). We used the original English version with Chinese instructions validated in prior studies. This unidimensional scale consists of 6 items, such as “Right now I feel satisfied with my physical appearance,” and uses a 9-point Likert scale. On this scale, 1 represents “very dissatisfied” and 9 represents “very satisfied,” with higher scores indicating greater satisfaction with one's body image. In this study, the BISS achieved an internal consistency coefficient of 0.89.

#### Restrained Eating Scale

The daily Restrained Eating Scale integrates items from the Eating Attitude Test (Garner et al., 1982) and the Eating Disorder Examination Questionnaire (Fairburn and Beglin, 1994). The Chinese composite version was developed by Wang et al. (2015). It comprises six items, such as “Today, I did not eat even when I was hungry,” and employs a 4-point Likert scale. On this scale, 1 signifies “not at all” and 4 signifies “completely,” with higher scores denoting a greater tendency toward restrictive eating behaviors. In this study, the scale demonstrated an internal consistency coefficient of 0.89. The DEBQ subscale was used for initial screening, while the daily Restrained Eating Scale captured dynamic within-person variations across the daily report period.

### Procedure

Initially, the DEBQ questionnaire was randomly distributed among female students at a university, resulting in 415 valid responses. From these, 114 participants were identified as restrictive eaters, defined as those scoring an average of 3 or higher on the Restrained Eating subscale (Wang, 2012). Participants' BMI was calculated using self-reported weight and height (*M* = 20.34, *SD* = 3.05), and two participants with a BMI >23.00 were excluded [BMI = weight (kg)/height (m)^2^]. Subsequently, participants' levels of trait mindfulness were measured using the Mindful Attention Awareness Scale (MAAS).

To effectively capture the dynamic changes in state mindfulness's impact on body image and restrictive eating behaviors, a daily report method was employed. Participants recorded their state mindfulness, body image, and restrictive eating behaviors over six consecutive days, ensuring comprehensive data collection. Participants received daily SMS reminders to submit electronic daily reports via a secure platform. Each diary entry included instructions to report their current state mindfulness, body image, and eating behaviors within the past 24 h.

Out of the 114 participants, 47 did not meet the data quality standards (defined as a diary completion rate below 50%). Of the remaining participants, 57% completed the 6-day questionnaire, and after excluding the two overweight participants, the final sample included 339 questionnaires from 65 female college students (37 participants completed the 6-day questionnaire, six participants completed 5 days, 21 completed 4 days, and one completed 3 days).

### Statistical analysis

The statistical analyses were conducted using SPSS 22.0 for inter-individual variables, including trait mindfulness, average state mindfulness, average body image, and average restrictive eating behaviors, by performing Pearson correlation analyses. Additionally, Mplus version 5.21 was used for intra-individual level correlation analyses and to test the multilevel mediation model of mindfulness, body image, and restrictive eating among female college students identified as having restrictive eating patterns (Zhang et al., 2016).

## Results

### Descriptive statistics

The Intraclass Correlation Coefficient (ICC) signifies the within-group correlation. The lower half of Table 1 presents the daily level correlation coefficients (*n* = 339), while the upper half contains the inter-individual average level correlation coefficients over 6 days (*n* = 65).

**Table 1:** Correlations between trait mindfulness, state mindfulness, body image, and restrictive eating.

**Variable**	***M***	***SD***	***ICC***	**2**	**3**	**4**
Trait mindfulness	3.85	0.68		0.51^**^	0.25^*^	−0.28^*^
State mindfulness	4.23	0.87	0.64			−0.38^**^
Body image	4.58	1.00	0.55	0.20^**^	0.41^**^	−0.43^**^
Restrictive eating	2.33	0.51	0.59	−0.42^**^	−0.30^**^	

^*^p <0.05, ^**^p <0.01.

Table 1 provides the means, standard deviations, ICC, and correlation coefficients for the variables. At the inter-individual level, trait mindfulness was significantly positively correlated with state mindfulness (*r* = 0.51, *p* < 0.01) and body image (*r* = 0.25, *p* < 0.05); state mindfulness was significantly positively correlated with body image (*r* = 0.41, *p* < 0.01); restrictive eating was significantly negatively correlated with trait mindfulness (*r* = −0.28, *p* < 0.05), state mindfulness (*r* = −0.38, *p* < 0.01), and body image (*r* = −0.43, *p* < 0.01). At the intra-individual level, state mindfulness was significantly positively correlated with daily body image (*r* = 0.20, *p* < 0.01); daily restrictive eating was significantly negatively correlated with state mindfulness (*r* = −0.42, *p* < 0.01) and daily body image (*r* = −0.30, *p* < 0.01).

The ICC value for mindfulness was 0.64, indicating that 64% of the variance was attributable to inter-individual differences in trait mindfulness, while 36% was due to intra-individual differences in state mindfulness. This supports the conceptualization of mindfulness as comprising both state and trait components, justifying the use of multilevel structural equation modeling.

### Regression analysis results

Table 2 presents the multilevel analysis results of state mindfulness, trait mindfulness, and restrictive eating behaviors. It was found that state mindfulness significantly negatively predicted restrictive eating behaviors at both the inter-individual and intra-individual levels (*p* < 0.001). At the inter-individual level, trait mindfulness also significantly negatively predicted restrictive eating behaviors.

**Table 2:** Predictive models of restrictive eating by state mindfulness and trait mindfulness.

**Variable**	**Independent variable: state mindfulness**		**Independent variable: trait mindfulness**	
	**Dependent variable: restrictive eating**		**Dependent variable: restrictive eating**	
	**Estimate**	***SE***	**Estimate**	***SE***
**Between-person**				
Intercept	1.52^***^	0.27	8.88^***^	1.87
Mindfulness	−0.76^***^	0.12	−0.12^***^	0.03
Residual	0.17^***^	0.03	6.73^***^	1.40
**Within-person**				
Mindfulness	−0.30^***^	0.04		
Residual	0.23^***^	0.03		

The model is a random intercept model; the average number of observations per person is 5.22, with an individual-level N = 65. The state mindfulness between individuals is the average score of the 6-day state mindfulness scale.

^*^p < 0.05, ^**^p < 0.01, ^***^p < 0.001.

These findings suggest that higher levels of both trait and state mindfulness are associated with lower tendencies toward restrictive eating behaviors, highlighting the potential of mindfulness interventions in addressing maladaptive eating patterns. The study's multilevel approach offers a comprehensive understanding of how mindfulness can influence body image and eating behaviors, providing valuable insights for developing targeted interventions aimed at improving mental health and wellbeing among female college students.

### Multilevel mediation analysis results

Table 3 presents the results of the multilevel mediation model examining how state mindfulness and trait mindfulness influence restrictive eating behaviors through body image.

**Table 3:** A multilevel mediation model of state mindfulness, trait mindfulness, restrictive eating, and body image.

**Variable**	**Mediation model (State mindfulness as the independent variable)**		**Mediation model (Trait mindfulness as the independent variable)**	
	**Dependent variable: restrictive eating**		**Dependent variable: restrictive eating**	
	**Estimate**	***SE***	**Estimate**	***SE***
**Between-person**				
Path a_b_	−0.12	0.20	0.12	0.10
Path b_b_	0.26	1.46	0.16	0.16
Path c_b_	−0.22	0.79	−0.03	0.19
Indirect effect	0.06	0.12	0.03	0.03
Residuals of the dependent variable	0.16	0.57	1.93	47.95
Residuals of the mediator variable	0.60	0.33	33.28^***^	10.66
**Within-person**				
Path a_w_	0.21^***^	0.06		
Path b_w_	0.31^***^	0.08	0.09	0.07
Path c_w_	0.02	0.05		
Indirect effect	0.66^***^	0.03		
Residuals of the Dependent Variable	0.22^***^	0.03	9.07^***^	1.35
Residuals of the mediator variable	0.29^***^	0.04		

The model is a random intercept model; the average number of observations per person is 5.22, with individual-level N = 65. (a_b_/a_w_) represents the path coefficient from mindfulness to body image; (b_b_/b_w_) represents the path coefficient from body image to restrictive eating behavior; (c_b_/c_w_) represents the path coefficient from mindfulness to restrictive eating behavior.

^*^p < 0.05, ^**^p < 0.01, ^***^p < 0.001..

The multilevel mediation model demonstrated acceptable fit indices: $\chi_{\left(12\right)}^{2}$ = 24.56, GFI = 0.90, CFI = 0.91, TLI = 0.93, RMSEA = 0.06, SRMR (within) = 0.04, SRMR (between) = 0.08. These indices suggest that the model adequately captured the relationships between variables at both intra- and inter-individual levels (Hu and Bentler, 1999).

**Figure 1:**
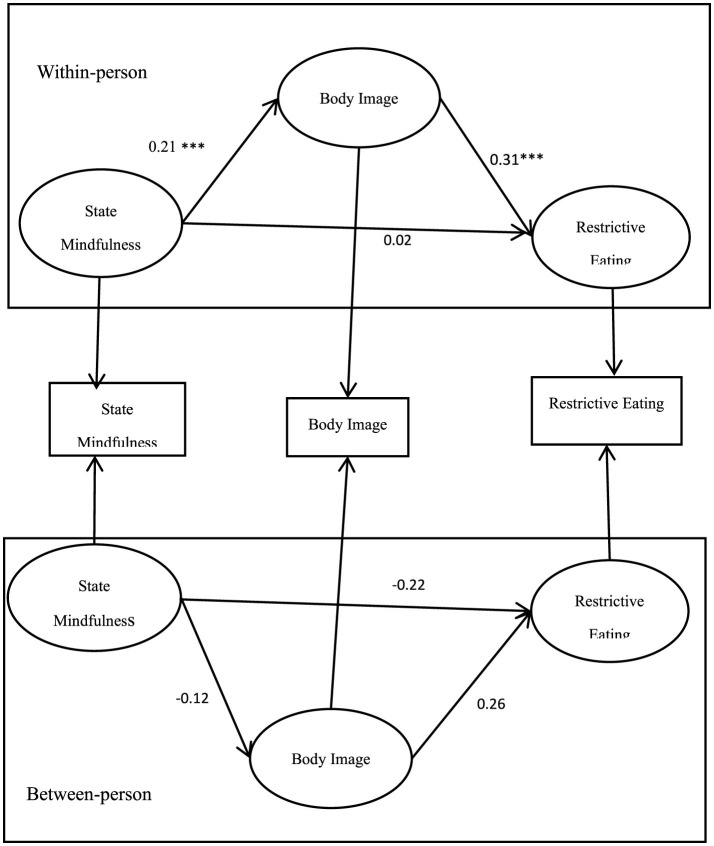
Diagram of the multilevel structural equation model for state mindfulness, body image, and restrictive eating. ^***^p < 0.001.

When state mindfulness was used as the predictor variable, at the inter-individual level, the path coefficients among state mindfulness, body image, and restrictive eating behaviors were not significant (*p* > 0.05). However, at the intra-individual level, the path coefficient from state mindfulness to body image (aw) was significant (*p* < 0.001), and the path coefficient from body image to restrictive eating behaviors (bw) was also significant (*p* < 0.001). The path coefficient from state mindfulness directly to restrictive eating behaviors (cw) was not significant (*p* > 0.05), as illustrated in Figure 1.

**Figure 2:**
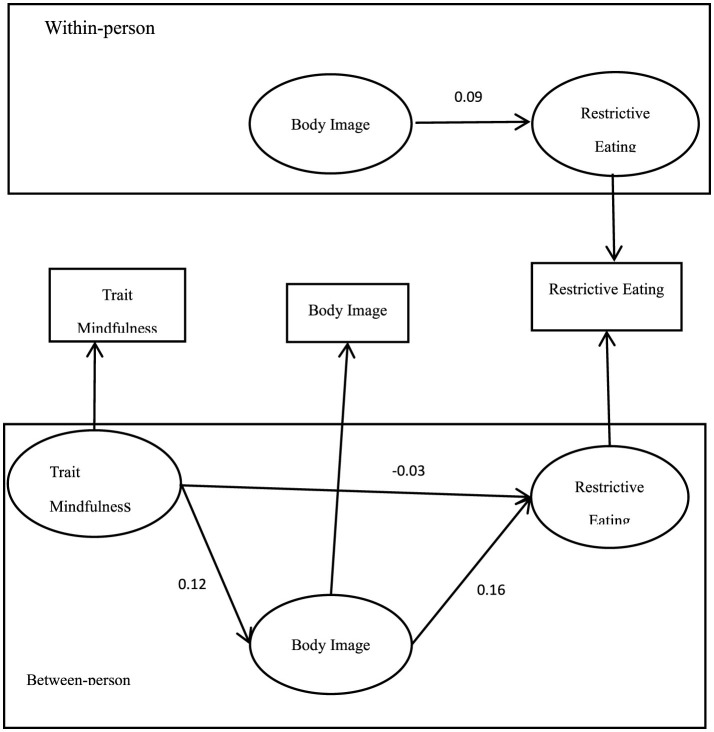
Diagram of the multilevel structural equation model for trait mindfulness, body image, and restrictive eating.

When trait mindfulness was used as the predictor variable, at the inter-individual level, the path coefficients among trait mindfulness, body image, and restrictive eating behaviors were not significant (*p* > 0.05), as shown in Figure 2.

These results indicate that at the intra-individual level, state mindfulness can significantly predict restrictive eating behaviors through body image, with body image serving as a full mediator in the relationship between state mindfulness and restrictive eating behaviors. This suggests that fluctuations in an individual's state mindfulness can lead to changes in how they perceive their body image, which in turn influences their eating behaviors. The absence of significant findings at the inter-individual level for both state and trait mindfulness highlights the importance of examining these variables in a dynamic, within-person context to fully understand their impact on eating behaviors.

The findings underscore the potential for interventions targeting state mindfulness to enhance body image perceptions and thereby reduce restrictive eating behaviors in real-time. This approach could be particularly beneficial in developing therapeutic strategies aimed at promoting healthier eating behaviors and improving psychological wellbeing among individuals who experience restrictive eating patterns.

## Discussion

This study employed a daily report method to investigate the effects of state and trait mindfulness on restrictive eating behaviors and explored the mediating role of body image. The findings provide novel insights into the relationships between mindfulness, body image, and restrictive eating across both inter- and intra-individual levels.

At the intra-individual level, state mindfulness significantly reduced daily restrictive eating behaviors, with body image serving as a full mediator. This suggests that when individuals experience higher levels of state mindfulness on a given day, they develop a more positive body image, which in turn reduces dietary restrictions. These results align with previous findings that mindfulness enhances body satisfaction and reduces maladaptive eating behaviors (Rodríguez et al., 2014; Kong et al., 2014). In contrast, at the inter-individual level, neither state nor trait mindfulness exerted indirect effects through body image. This discrepancy highlights the situational nature of body image and restrictive eating behaviors, which are prone to daily fluctuations (Cash et al., 2002).

The significant correlations observed between mindfulness, body image, and restrictive eating behaviors are consistent with prior studies (Breines et al., 2014; Kelly and Stephen, 2016). However, our findings differ from Ouwens et al. (2015), who reported a positive relationship between trait mindfulness and restrictive eating among individuals with obesity. This discrepancy may stem from differences in study samples: while overweight individuals may use restrictive eating to limit excessive intake, restrictive eaters within a normal BMI range often limit necessary dietary intake. This highlights the importance of considering sample-specific contexts in future research.

The mechanisms underlying these relationships can be explained by mindfulness's role in enhancing interoceptive awareness. For restrictive eaters, state mindfulness may reduce habitual negative evaluations of body image, facilitating more adaptive eating behaviors. However, unlike general populations, restrictive eaters exhibit attentional and memory biases toward food and body-related cues (Kong et al., 2011). According to cognitive-behavioral theory, altering these maladaptive cognitive patterns is essential for behavioral change. This explains why state mindfulness alone cannot directly reduce restrictive eating but operates through improvements in body image. State mindfulness may enhance interoceptive awareness by reducing cognitive fusion with negative body-related thoughts (Fisher et al., 2022), thereby interrupting the cycle of body dissatisfaction and restrictive eating.

From a practical perspective, these findings underscore the potential of state mindfulness as an intervention target. Compared to trait mindfulness, state mindfulness is more malleable and can be enhanced through mindfulness-based training programs. By fostering an accepting attitude toward body perceptions, state mindfulness can reduce excessive dietary restrictions and promote healthier eating patterns. Additionally, cultivating state mindfulness may facilitate the development of trait mindfulness over time, providing long-term benefits for eating disorder prevention (Hülsheger et al., 2013).

However, the lack of mediation effects at the inter-individual level raises important questions. It is possible that relatively stable traits like mindfulness are less influenced by situational variables such as daily body image or restrictive eating behaviors. Future studies should examine whether other stable personality factors, such as self-esteem, mediate these relationships (Kelly and Stephen, 2016).

### Limitations

This study has several limitations. First, the sample was restricted to female students from two universities in Guangxi, China, which may limit generalizability to populations with diverse cultural, socioeconomic, or gender backgrounds. Second, although the sample size met simulation-based guidelines for within-person effects (Zhang et al., 2016), the between-person sample (*N* = 65) may lack power to detect smaller inter-individual effects, particularly for trait mindfulness. Third, self-reported measures (e.g., dietary behaviors, mindfulness) are susceptible to recall and social desirability biases; future studies should incorporate objective measures (e.g., ecological momentary assessment, physiological biomarkers). Fourth, the exclusion of participants with BMI >23.00 may inadvertently omit individuals with subclinical eating disorders, limiting insights into higher-weight populations. Finally, potential confounding variables (e.g., menstrual cycle phases, academic stress) were not controlled, which could influence daily body image and eating behaviors.

## Conclusion

Despite these limitations, this study highlights the nuanced relationship between mindfulness, body image, and restrictive eating. By integrating intra- and inter-individual perspectives through a diary design, we demonstrate that state mindfulness reduces restrictive eating behaviors via improved body image at the within-person level. These findings underscore the potential of real-time mindfulness interventions (e.g., mobile-based mindfulness training) to disrupt the cycle of body dissatisfaction and maladaptive eating patterns among young women. Future research should extend these findings to clinical populations and diverse cultural contexts to advance targeted therapeutic strategies.

## Data Availability

The raw data supporting the conclusions of this article will be made available by the authors, without undue reservation.
